# Brain Tumor Stem Cell Multipotency Correlates with Nanog Expression and Extent of Passaging in Human Glioblastoma Xenografts

**DOI:** 10.18632/oncotarget.1059

**Published:** 2013-06-08

**Authors:** Dominique M. Higgins, Ruisi Wang, Brian Milligan, Mark Schroeder, Brett Carlson, Jenny Pokorny, Samuel H. Cheshier, Fredric B. Meyer, Irving L. Weissman, Jann N. Sarkaria, John R. Henley

**Affiliations:** ^1^ Medical Scientist Training Program, Mayo Clinic: College of Medicine, Rochester, Minnesota, USA; ^2^ Mayo Graduate School, Mayo Clinic: College of Medicine, Rochester, Minnesota, USA; ^3^ Department of Neurologic Surgery, Mayo Clinic, Rochester, MN, USA; ^4^ Department of Radiation Oncology, Mayo Clinic, Rochester, MN, USA; ^5^ Department of Neurosurgery, Stanford University School of Medicine, Stanford, CA, USA; ^6^ Institute for Stem Cell Biology and Regenerative Medicine and the Ludwig Cancer Center, Stanford University Medical Center, Stanford, CA

**Keywords:** nanog, glioma, GBM, brain tumor stem cell, multipotency, xenograft, SDF-1

## Abstract

Glioblastoma multiforme (GBM) is the most common primary brain tumor, with a median survival of only 15 months. A subpopulation of cells, the brain tumor stem cells (BTSCs), may be responsible for the malignancy of this disease. Xenografts have proven to be a robust model of human BTSCs, but the effects of long-term passaging have yet to be determined. Here we present a study detailing changes in BTSC multipotency, invasive migration, and proliferation after serial passaging of human GBM xenografts. Immunocytochemistry and tumorsphere formation assays demonstrated the presence of BTSCs in both early generation (EG-BTSCs; <15 passages) and late generation (LG-BTSCs; >24 passages) xenografts. The EG-BTSCs upregulated expression of lineage markers for neurons and oligodendrocytes upon differentiation, indicating multipotency. In contrast, the LG-BTSCs were restricted to an astrocytic differentiation. Quantitative migration and proliferation assays showed that EG-BTSCs are more migratory and proliferative than LG-BTSCs. However, both populations respond similarly to the chemokine SDF-1 by increasing invasive migration. These differences between the EG- and LG-BTSCs were correlated with a significant decrease in nanog expression as determined by qRT-PCR. Mice implanted intracranially with EG-BTSCs showed shorter survival when compared to LG-BTSCs. Moreover, differentiation prior to implantation of EG-BTSCs, but not LG-BTSCs, led to increased survival. Thus, nanog may identify multipotent BTSCs. Furthermore, limited passaging of xenografts preserves these multipotent BTSCs, which may be an essential underlying feature of GBM lethality.

## INTRODUCTION

Glioblastoma multiforme (GBM) is a grade IV astrocytoma and is the most common primary CNS brain tumor [1-3]. The cells are highly malignant and invasive, and currently there exists no cure. Despite aggressive surgery, chemotherapy, and radiotherapy, these tumors inevitably recur and result in death [1, 3, 4]. Cancer stem cells have been implicated in not only the origin of GBM [5-8], but also its refractory nature to treatment [9]. As such, targeted therapy towards this subpopulation of cells provides potential for significant improvement in outcomes [7]. One defining aspect of these cells is their ability to differentiate into lineage specific cells, or multipotency. In GBM, the antigens CD133 and nestin have been shown to enrich for brain tumor stem cells (BTSCs) [5, 10-13]. Recently, expression of the transcription factor nanog, which is required for the pluripotency of embryonic stem cells, has also been shown to positively correlate with astrocytoma malignancy and self-renewal capability of BTSCs [14-20].

Mounting evidence has demonstrated that the tumor microenvironment plays a crucial role in the regulation of GBM growth and progression via growth factors, angiogenic factors, and chemotactic cues [21-25]. In particular, stereotypical patterns of GBM invasion, involving migration along blood vessels and axons, have been well described and are highly correlated with the expression of chemokine ligands and receptors [26-28]. Of note are the chemokine stromal derived factor-1 (SDF-1, or CXCL12), and its receptor CXCR4 [27]. The SDF-1/CXCR4 axis is primarily known for its role in regulating physiologic immune cell migration [29-35]. However, it is now known that CXCR4 is highly expressed in multiple cancers, including gliomas [36-42]. The ligand SDF-1 is expressed by endothelial cells of blood vessels, neurons, and other common migratory pathways in the brain, and promotes GBM invasion [27, 39, 43-45].

Several models have been developed to study GBM and other cancers in order to better understand how stem cells might contribute to the pathology [46]. Long-term passaged cell lines have been used primarily for *ex vivo* modeling. However, with accumulating evidence supporting the role of cancer stem cells in tumor malignancy, and evidence of distinct GBM subtypes [47, 48], models more closely resembling the ‘parent’ tumor have become necessary [49-51]. An attractive alternative is the use of primary xenograft tumors [21, 46, 52, 53]. These tumors are established by direct implantation of resected tumor specimens from patients and passaged in the flanks of immunocompromised mice. Passaging tumor cells by this method has been shown to preserve the original mutation status of the parent tumor [21, 46, 49, 50]. One critical question regarding this system, however, is whether extended passaging influences the multipotency and invasiveness of the BTSCs. The ability to passage these xenograft lines indefinitely without affecting the biology of the stem cell population would be advantageous from a practical standpoint. In addition, an understanding of the evolution of these tumors could potentially provide insight into the pathophysiology of tumor formation and recurrence.

To this end, we investigated how serial passaging affects GBM BTSCs in a xenograft model. We have found that limited passaging of xenografts, as compared to extended passaging, preserves multipotency, invasive migration *ex vivo*, and lethality *in vivo*. Intriguingly, these characteristics correlate positively with elevated nanog expression. In addition, the functional data demonstrate that chemokinetic responses to SDF-1 are maintained despite extended passaging.

## RESULTS

### Early and late generation xenografts possess GBM stem cells

**Figure 1:**
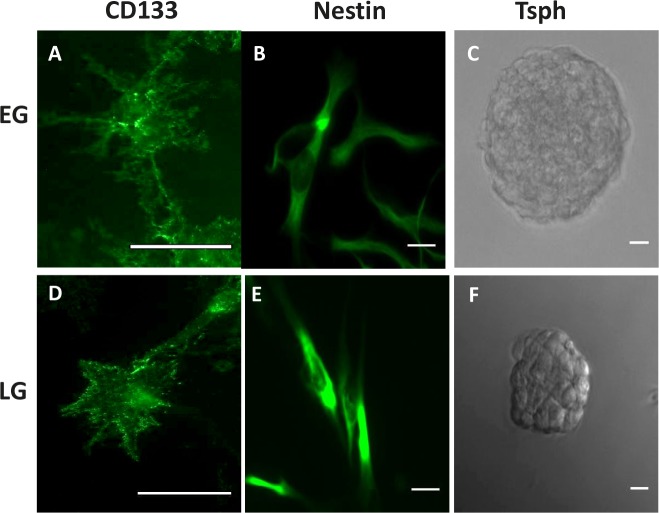
GBM Xenografts possess BTSCs Immunocytochemistry demonstrating CD133 and nestin expression in EG- (A, B) and LG-BTSCs (D,E). Phase-contrast microscopy of tumorspheres (C, F). (Scale bar = 25 μm)

First, we determined whether limited passaging (< 15 passages following initial implantation; Early Generation) of xenografts preserves the BTSCs, and conversely, whether extended passaging (>24 passages; Late Generation) for greater than 2 years, would deplete the BTSCs. Immunostaining xenograft cells that were grown in stem cell conditions revealed that both early generation (EG) and late generation (LG) xenografts expressed the stem cell markers CD133 and nestin (Fig. 1A,B,D,E). Additionally, both EG and LG xenografts were capable of forming self-adherent balls of tumor cells known as tumorspheres when plated in stem cell conditions in the absence of extracellular matrix, indicative of the presence of tumor stem cells (Fig. 1C,F).

### Limited passaging of xenografts preserves multipotency

**Figure 2:**
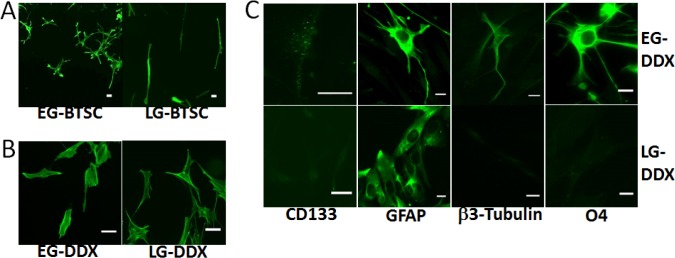
EG-BTSCs are multipotent (A) Fluorescent phalloidin staining demonstrating differences in morphology between EG- and LG-BTSCs (green). EG-BTSCs are multipolar and astrocytic, whereas LG-BTSCs are bipolar and fibroblastic. (B) Both EG differentiated (EG-DDX) cells and LG differentiated (LG-DDX) cells stained with phalloidin demonstrate changes in morphology with differentiation. (C) Immunofluorescence analysis of CD133 and lineage marker expression in EG- and LG-DDX cells. Both EG and LG-DDX cells lose CD133 expression and express the astrocytic marker GFAP (green). Only EG-DDX cells upregulate β3-tubulin (neuronal) and O4 (oligodendrocytic) (green). Scale bar = 25 μm.

Despite the presence of stem cells in both populations, we observed that LG cells cultured in stem cell media (LG-BTSCs) had a distinct morphology when compared to EG cells grown in identical conditions (EG-BTSCs). Whereas the EG-BTSCs tended to be more multipolar and astrocytic in appearance, LG-BTSCs were predominantly bipolar, as revealed by staining for filamentous actin (Fig. 2A). To determine if the morphologic changes correlated with changes in multipotency, stem cells were assayed for their ability to differentiate in serum-containing media. Fluorescence microscopy of serum-treated cells showed that both EG- and LG-BTSCs changed morphology (Fig. 2B) and lost CD133 expression (Fig. 2C), consistent with differentiation. Immunostaining to detect markers for neurons (β3-tubulin), astrocytes (GFAP), and oligodendrocytes (O4) determined the ability of the stem cells to differentiate into the 3 neural lineages. Following the differentiation-treatment, the EG-BTSCs demonstrated a concomitant increase in expression of all three lineage markers, β3-tubulin, GFAP, and O4, indicating multipotency (Fig. 2C). In contrast, the differentiated LG-BTSCs expressed GFAP, (Fig. 2C), but not β3-tubulin or O4, indicating a loss of multipotency.

### EG and LG-BTSC migration is promoted by SDF-1

**Figure 3:**
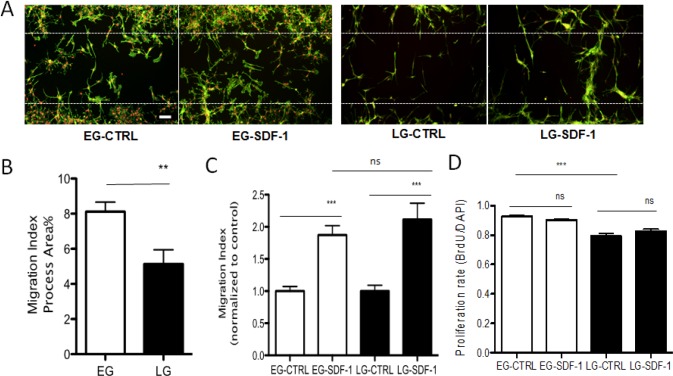
EG-BTSCs are more migratory and proliferative than LG-BTSCs (A) Immunostaining of gap migration assay (phalloidin=green; DAPI=red) and (B) quantitative analysis showing BTSC invasive migration into the gap (white dashed line indicates boundary of gap). EG-BTSCs demonstrate increased invasive migration compared to LG-BTSCs. Migration index was 8.1% for EG-BTSCs, and 5.1% for LG-BTSCs. (C) EG- and LG-BTSCs have a similar magnitude response to chemotrophic signaling. Migration index of SDF-1 treated stem cells filling in the gap, as normalized to control, was 1.9 for EG-BTSCs, and 2.1 for LG-BTSCs. (D) BrdU labeling demonstrating EG-BTSCs are more proliferative than LG-BTSCs (0.93 vs. 0.79, respectively). Treatment with SDF-1 had no effect on the proliferation rate of either population. (**=p< 0.01; ***=p< 0.001; data from at least 3 independent experiments). Scale bar = 100 μm).

Given the differences in multipotency of the two populations of BTSCs, we evaluated whether or not this correlated with differences in invasiveness by comparing baseline rates as well as responsiveness to chemotactic cues present in the tumor microenvironment. Both EG- and LG-BTSCs expressed the SDF-1 ligand and receptors CXCR4 and CXCR7, as determined by PCR analysis (Supp Fig. 1). Using a quantitative cell-based migration assay, we compared baseline migration between EG- and LG-BTSCs. Briefly, BTSCs were plated onto coverslips around an inserted barrier that created a cell free gap. At time zero, the insert was removed. At the 48-hour end point, cells were fixed and stained for F-actin to label cellular processes. Fluorescence microscopy and quantitative analysis determined the percentage of cells that had migrated into the gap, or the migratory index. The EG-BTSCs had a significantly increased migratory index compared to LG-BTSCs (8.1% ± 0.5, mean± SEM vs. 5.1% ± 0.8, respectively) (Fig. 3A,B). Given the prominent role of SDF-1 in GBM invasion [39], we compared the migratory response of EG- and LG-BTSCs to this chemokine. Both EG- and LG-BTSCs displayed a similar magnitude increase in migratory index (1.9 fold ± 0.1 SEM vs. 2.1 fold ± 0.3, respectively) during SDF-1 treatment (added at time zero) (Fig. 3A,C). Thus, EG-BTSCs are intrinsically more migratory than LG-BTSCs, but both show similar responsiveness to SDF-1.

### EG-BTSCs are more proliferative than LG-BTSCs

Previous studies have implicated a role for SDF-1 in regulating proliferation of GBM cells [36]. We therefore compared the intrinsic proliferation rates of EG- and LG- BTSCs and in response to SDF-1. Labeling with BrdU, an indicator of cell division, followed by quantitative fluorescence microscopy showed that EG-BTSCs had a greater percentage of BrdU positive cells compared to LG-BTSCs (93% ± 0.6 SEM vs. 79% ± 1.8 BrdU positive, respectively) (Fig. 3D). However, treatment with SDF-1 had no effect on BrdU labeling of either population (Fig. 3D). Thus, we conclude that EG-BTSCs are more proliferative than LG-BTSCs, and SDF-1 has no effect on the proliferation rate of either.

### Nanog expression is elevated in multipotent EG-BTSCs

**Figure 4:**
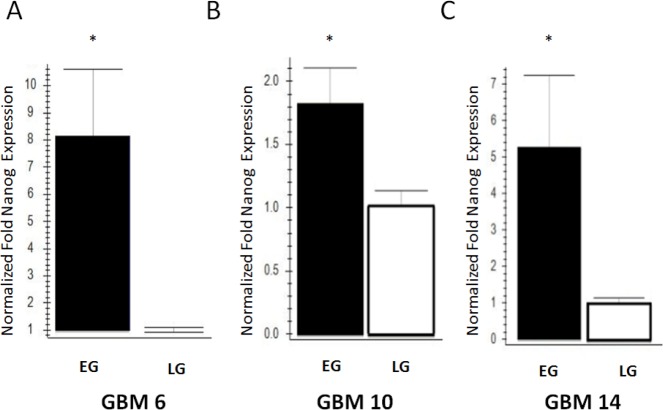
Increased nanog expression in EG-BTSCs compared to LG-BTSCs cDNA obtained from flank tumors was used for qRT-PCR analysis of nanog mRNA expression, using actin has a housekeeping gene. Nanog was consistently elevated across 3 different human GBM xenograft lines, (A) GBM6 (8.2-fold), (B) GBM10 (1.8-fold), and (C) GBM14 (5.3-fold). (n=3, *=p<0.05)

Based on our finding that EG-BTSCs, but not LG-BTSCs are multipotent, we examined the expression of *nanog*, a known multipotency gene. Analysis by qRT-PCR demonstrated that nanog mRNA expression levels were significantly higher in EG tumors compared to LG tumors in the 3 out of 3 human GBM xenograft lines tested, GBM6 (8.2-fold ± 2.4 SEM), GBM10 (1.8-fold ± 0.3), and GBM14 (5.3-fold ± 2.0) (Fig. 4A,B,C, p<0.05 for each line). This difference was therefore present across xenograft lines of variable genetic backgrounds, and thus may be a general feature of EG-BTSCs. The most dramatic difference was found in GBM6, an EGFRviii mutant tumor, followed by GBM10 and GBM14, which both harbor PTEN mutations but wild type EGFR.

### Differential effects of EG- and LG-BTSCs *in vivo*

**Figure 5:**
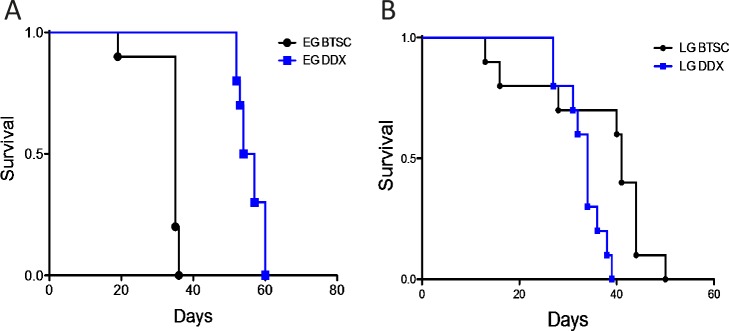
EG-BTSCs are more lethal and differentiation sensitive than LG-BTSCs (A) EG-BTSCs and EG-DDX cells were stereotactically injected intracranially into athymic nude mice, which were then observed for survival times. EG-BTSCs (black circles) demonstrated a much shorter survival time than EG-DDX cells (blue squares) (n=10, p<0.0001). (B) LG-BTSCs (black circles) did not lose tumorigenicity on differentiation, as LG-DDX (blue squares) cells resulted in an even shorter survival time (n=10, p<0.01)

To test whether EG and LG GBM cells displayed different characteristics *in vivo*, each was briefly cultured and injected intracranially into athymic nude mice to determine the length of time to reach a moribund state (survival time). Because previous studies have shown that GBM stem cells are more tumorigenic than their non-stem cell counterparts [11, 54], we compared survival in animals injected with differentiated cells derived from either EG or LG xenografts. Animals injected with differentiated EG GBM cells (EG-DDX) had a significantly increased median survival time (55.5 days, n=10) compared to that of EG-BTSCs (35 days, n=10), consistent with the notion that differentiated cells are less tumorigenic (Fig. 5A). In contrast, there was no such increase in survival of mice injected with differentiated LG GBM cells (LD-DDX) (34 days, n=10) compared to the lineage restricted LG-BTSCs (41 days, n=10) (Fig. 5B). Comparing the BTSC cohorts, the LG had an increased survival time relative to the EG (p<0.05) (Fig. 5A,B). Taken together, these data indicate that the EG-BTSCs are more lethal and differentiation sensitive than the LG-BTSCs.

**Table 1:** Comparison of Early and Late Generation BTSC characteristics

	EG-BTSC	LG-BTSC
Stem Cell Markers	Positive	Positive
Tumorsphere Formation	Positive	Positive
Multipotency	3 lineages	lineage restricted
Nanog Expression	High	Low
Invasiveness	High	Low
Chemokine Response	Positive	Positive
Proliferation	High	Low

## DISCUSSION

Here, we investigated how extended passaging of GBM xenografts affects BTSCs. Our results indicate that whereas brief passaging preserves multipotency of BTSCs, extended passaging results in a loss of multipotency with a corresponding decrease in cell migration, proliferation, and lethality. Additionally, we show that maintenance of BTSC multipotency correlates with relatively high levels of nanog expression.

Inducing differentiation of EG-BTSCs produced cells resembling all three neural lineages; that is, astrocytic, neuronal and oligondendrocytic, confirming multipotency. However, differentiation of LG-BTSCs gave rise only to astrocytic cells, which indicates lineage restriction. Potentially, this phenotypic change during progression from EG- to LG xenografts is either the result of genetic drift of the entire population (eg. acquired mutations or epigenetic changes), or the selection of a subpopulation of cells. The LG cells cultured in stem cell conditions may therefore represent a progenitor cell derived from the EG-BTSC. Future investigations into the natural evolution of BTSC populations within GBM may provide significant new insight into the origin of tumor initiating cells, as well as advance our understanding of tumor recurrence.

Recent studies have provided compelling evidence for the role of nanog in maintenance of BTSC proliferation and multipotency [14-20]. For example, expression of *nanog* and other signature embryonic stem cell genes was associated with more aggressive tumors and poorer prognoses in several cancers, including GBM [20]. Additionally, the microRNA cluster miR 302-367 was sufficient to suppress the stem cell phenotype of glioma-initiating cells and decreased nanog expression [17]. In U87 cell lines, nanog inhibition by miR-134 was sufficient to decrease proliferation and invasion [16, 19]. In accordance with these findings, we have demonstrated in the present study that low nanog expressing GBM xenografts display a loss of multipotency, as well as diminished proliferation and invasive migration. Further studies are now warranted to demonstrate whether or not nanog expression is sufficient to drive the phenotypic differences seen between EG and LG-BTSCs.

Expression analysis has defined at least 3 subtypes of GBM, including Proneural, Classical, and Mesenchymal [48]. These classifications have also been validated in human GBM xenografts [48]. Alterations in EGFR, particularly the viii mutation such as in GBM6, correlates highly with the classical subtype. The PTEN mutations present in GBM10 and GBM14 were most often found in the Mesenchymal subtype. Thus, our finding correlating the relationship of nanog expression with the multipotency of BTSCs is maintained across tumors of varying genetic backgrounds, and potentially across multiple GBM subtypes.

Previous studies comparing *in vivo* characteristics of GBM stem cells versus non-stem cells have shown that the former have a significantly increased tumorigenic capacity, and consequently result in shorter survival times of implanted mice [11, 54-56]. Our finding that differentiating the multipotent EG-BTSCs correlated with decreased tumorigenicity and increased survival is consistent with these previous reports. Moreover, our result that inducing differentiation of the lineage restricted LG-BTSC, which caused no decrease in tumorigenicity or survival, may be attributable to an inability of the LG-BTSCs to differentiate.

In contrast to a previous report that GBM stem cells are stimulated to proliferate by SDF-1 [36], we found no changes in BTSC proliferation in response to SDF-1 in either EG- or LG models. This incongruence may be explained by the different models used. Whereas in the present study we passaged tumor cells as xenografts, the previous study utilized GBM stem cells maintained in *ex vivo* cultures. Thus, BTSC proliferative responsiveness to SDF-1 may be contingent upon the stem cell model employed.

Xenografts have proven to be a useful model for cancer research, enabling significant molecular insights into the biology of tumors, and the role of chemotherapeutics. Our work demonstrates the importance of limited passaging in order to study cancer stem cells in a xenograft model. Moreover, extended passaging may represent later stages in the spectrum of cancer development, and as such may be valuable for studying other aspects of this disease, like progression and recurrence.

## MATERIALS AND METHODS

### Establishment of GBM Xenografts

Primary GBM samples were resected from patients and subsequently used for direct implantation into the flank of 6-8 week old athymic nude mice in accordance with National Institutes of Health (NIH, Bethesda, MD, USA) and the Mayo Clinic (Rochester, MN, USA) Institutional Review Board and Institutional Animal Care and Use Committee guidelines. Briefly, a 1:1 ratio by volume of tumor and Matrigel (Fisher) was mixed and injected subcutaneously into athymic nude-foxn1^nu^ mice (Harlan) flanks. Each month, flank tumors were resected and either utilized for *ex vivo* assays, preserved for histopathologic and molecular analysis, cryopreserved for future tumor re-establishment, or serially passaged into another mouse. Early generation (EG) tumors were defined as those passaged less than 15 times, whereas late generation (LG) tumors were passaged greater than 24 times.

### Brain Tumor Stem Cell Culture

Flank GBM xenografts (GBM6, 10, 14) were allowed to grow for 4-6 weeks according to institutional guidelines. Tumors were then carefully dissected away from the mouse using a No. 10 blade scalpel, and placed into PBS with antibiotics. Shortly after, the tumor capsules were carefully opened and tumor was stripped away using forceps. The samples were then mechanically dissociated with a scalpel and large, visible blood vessels were removed using forceps. This was followed by enzymatic dissociation with 160 units of papain (Worthington) in dPBS (Life Tech) with DNAse I (Worthington) and L-cysteine (Sigma) for 30 minutes at 37^°^C. Dissociated cells were then centrifuged and transferred to ovomucoid (Worthington)/BSA (Sigma) solutions to stop the reaction. Cell solutions were then filtered using a 40μm mesh filter (Nitex). Dissociated cells were cultured in stem cell media comprised of Neurobasal A (Life Tech), non-essential amino acids (Life Tech), sodium pyruvate (Life Tech), penicillin/streptomycin (Life Tech), epidermal growth factor (20 ng/ml) (Sigma) and basic fibroblast growth factor (20 ng/mL) (Stem Cell Tech), and B27 supplement without vitamin A (Life Tech). Cells were plated as a monolayer on Matrigel coated 10 cm tissue culture dishes or as tumorspheres in 10 cm petri dishes at 37^°^C and 5% CO_2_. Differentiation was accomplished by withdrawal of the above stem cell growth factors and continued culture in DMEM (Life Tech) containing 10% FBS (Atlanta Biologicals) and penicillin/streptomycin.

### Immunocytochemistry

GBM6 cells were fixed with 4% paraformaldehyde, permeabilized with 0.1% Triton-X, and blocked with 10% normal goat serum (NGS) (Jackson ImmunoResearch). Cells were then stained with primary antibodies (5-10 μg/mL) anti-CD133 (Miltenyi), anti-nestin (Millipore), anti-GFAP (Abcam), anti-beta-3 tubulin (Abcam), or anti-O4 (Abcam), followed by washes with PBS, and incubation with goat anti-rabbit or goat anti-mouse Alexa 488 secondary antibodies (2 μg/mL, Life Tech). Imaging was done using a Zeiss Apotome microscope.

### Gap Migration Assay

GBM6 stem cells were dissociated from monolayer cultures using Tryple Express (Life Tech), and the reaction was stopped using Ovomucoid Trypsin Inhibitor (Worthington). Coverglasses (Carolina Bio) were sterilized by ethanol and UV treatment. Dried coverglasses were then pre-coated with poly-D-lysine (10 μg/mL, Sigma), followed by fibronectin (40 μg/mL, Sigma). Dissociated cells were plated onto fibronectin-coated coverslips in 24-well plates around a gap insert (Cell Bio Labs) at a density of 75,000 cells per well in duplicate. Cells were counted using a hemocytometer and trypan blue to exclude dead cells. After two days of culture, inserts were removed, and debris was washed away, leaving a cell-free gap. Cells were treated for 48 hours, and fixed with 4% paraformaldehyde. They were then stained with Alexa 488 conjugated Phalloidin (Life Tech) and DAPI after permeabilization with 0.1% Triton-X (Thermo Scientific) to label intracellular F-actin and nuclei. Coverslips were mounted using Prolong Gold (Life Tech) and imaged with the Zeiss AxioImager microscope. Analysis was conducted using ImageJ Software. Time zero images were used to evaluate consistency of gap sizes. To evaluate extent of migration, images were thresholded, and percent area of the cell free gap invaded was quantitated to determine the migratory index.

### Proliferation Assay

Dissociated GBM6 stem cells were plated onto fibronectin-coated coverslips as above. After 2 days, debris was washed away and cells were treated and pulsed with bromo-deoxy-uridine (BrdU) (Roche) for 48 hours. Cells were fixed with 70% acidic ethanol and stained with anti-BrdU antibody, followed by secondary anti-mouse Alexa 488 (Life Tech) and DAPI. Coverslips were then imaged using a Zeiss Apotome at 10x magnification of 13 representative fields. BrdU positive and DAPI positive cells were then counted.

### PCR Analysis

1-2 million cells were used for RNA isolation using the RNEasy Isolation Kit (Qiagen). RNA concentration and purity was evaluated using a spectrophotometer and reverse transcribed (Select cDNA Synthesis Kit, BioRad). Primers (Sigma) were used to perform PCR (Platinum PCR Supermix High Fidelity, Life Tech) or qRT-PCR (IQ Sybr Green Supermix, BioRad). Amplicons were analyzed by agarose DNA gel electrophoresis. qRT-PCR data was analyzed using the ∆∆Cq method.

### Primers

CXCR4 (Invitrogen):
F 5' - GGCCCTCAAGACCACAGTCA
R 5' -TTAGCTGGAGTGAAAACTTGAAG
CXCR7 (Invitrogen):
F 5'-TGGTCAGTCTCGTGCAGCAC
R 5'-GCCAGCAGACAAGGAAGACC
SDF-1 (Invitrogen):
F 5' - ATGAACGCCAAGGTCGTGGTC
R 5' -GGTCTGTTGTGCTTACTTGTTT
Nanog (IDT):
F 5' -GATTTGTGGGCCTGAAGAAA
R 5' -TTGGGACTGGTGGAAGAATC
Actin (Qiagen)
QuantiTect Primer Assay

### Intracranial GBM Implantation

Following short-term culture of GBM xenografts in stem cell media or differentiation media, GBM stem cells and differentiated cells, respectively, were harvested for intracranial injections in athymic nude mice. 300,000 cells per mouse were stereotactically injected according to existing protocols [53]. Mice were then observed daily for signs of neurologic decline and morbidity, at which point, they were euthanized.

### Statistics

Statistical analyses were performed using Graphpad Prism software (v5, La Jolla, CA, USA). The D'Agostino & Pearson omnibus test was used to assess normality of the data. Normally distributed experimental results were analyzed using the unpaired 2-tailed student's t-test for groups of 2, or one-way ANOVA with Bonferroni's post test for groups of more than 2. Nonparametric results were analyzed by either the Mann Whitney test (groups of 2) or Kruskal-Wallis with Dunn's post test (>2). Survival was analyzed using a Kaplan Meier Curve with a Log-rank (Mantel Cox) test.

## Supplementary Figures


