# Supplementary material for: Brain Tumor Stem Cell Multipotency Correlates with Nanog Expression and Extent of Passaging in Human Glioblastoma Xenografts
Source: Oncotarget. 2013 Jun 8;4(5):792–801. doi: 10.18632/oncotarget.1059 (PMC3742839; doi:10.18632/oncotarget.1059)
Supplement: Supplementary file 1 [file oncotarget-04-792-s001.pdf]

www.impactjournals.com/oncotarget/ Oncotarget, Supplementary Materials 2013

Brain Tumor Stem Cell Multipotency Correlates with Nanog Expression and Extent of Passaging in Human Glioblastoma Xenografts - Higgins et al

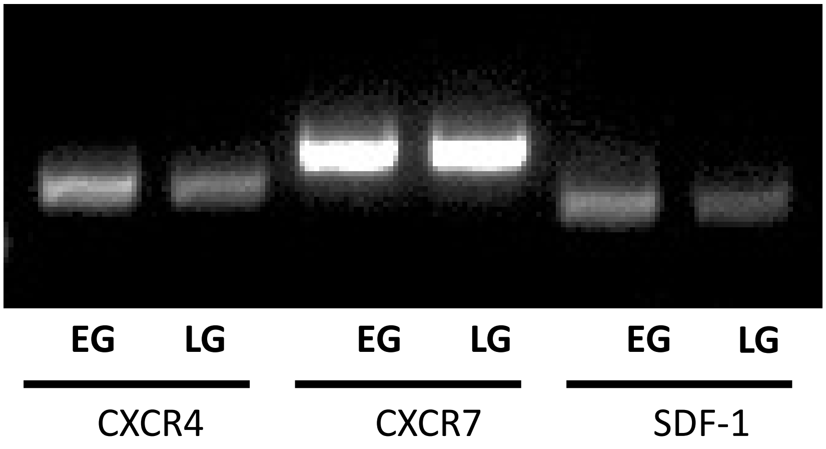

Supplementary Figure 1: PCR analysis demonstrating expression of CXCR4, CXCR7 and SDF-1 in EG- and LG-BTSCs.
